# The impact of depression on survival of head and neck cancer patients: A population-based cohort study

**DOI:** 10.3389/fonc.2022.871915

**Published:** 2022-08-25

**Authors:** Ren-Wen Huang, Kai-Ping Chang, Filippo Marchi, Charles Yuen Yung Loh, Yu-Jr Lin, Chee-Jen Chang, Huang-Kai Kao

**Affiliations:** ^1^ Department of Plastic and Reconstructive Surgery, Chang Gung Memorial Hospital at, Linkou, Taiwan; ^2^ Otolaryngology–Head and Neck Surgery, Chang Gung Memorial Hospital at, Linkou, Taiwan; ^3^ Clinical Informatics and Medical Statistics Research Center, Chang Gung University, Taoyuan, Taiwan; ^4^ Department of Plastic Surgery, Addenbrookes Hospital, Cambridge, United Kingdom

**Keywords:** depression, head and neck cancer, overall survival, population-based study, Taiwan

## Abstract

**Background:**

Depression is common among patients with head and neck cancer, thereby affecting their survival rate. However, whether close monitoring of depression affects the survival outcomes of these patients is unknown. Therefore, this study aimed to determine whether depression treatment continuity after the diagnosis of cancer affects the survival of these patients.

**Methods:**

A total of 55,069 patients diagnosed with head and neck cancer in the Cancer Registration System database in Taiwan were enrolled. This cohort was followed from January 1, 2007 to December 31, 2017. Furthermore, the patients were divided into four groups, namely, “no depression,” “pre-cancer only,” “post-cancer only,” and “both before and after cancer,” on the basis of the diagnosis of depression and the duration of the follow-up period in the psychiatric clinic. Further, the Cox proportional hazard model was applied to estimate the hazard of death for the four groups.

**Results:**

A total of 6,345 (11.52%) patients were diagnosed with depression in this cohort. The “pre-cancer only” group had a lower overall survival (HR = 1.18; 95% CI = 1.11–1.25) compared with the “no depression” group. Moreover, the “post-cancer only” group had better overall survival (HR = 0.88; 95% CI = 0.83–0.94) compared with the “no depression” group, especially in advanced-stage patients. Patients who were diagnosed with depression before cancer and had continuous depression treatments after the cancer diagnosis had better overall survival (HR = 0.78; 95% CI = 0.71–0.86) compared with patients who had treatment interruptions.

**Conclusion:**

Patients with pre-cancer depression had poorer survival outcomes, especially those who did not receive psychiatric clinic visits after their cancer diagnosis. Nonetheless, in patients with advanced-stage cancer, depression treatment may improve overall survival.

## Introduction

Depression is common in patients with cancer, and there might be possible links between depression and many cancers (1, 2). A meta-analysis review article has demonstrated a positive correlation between depression and cancer, especially in patients with lung and liver cancer (3). A nationwide matched cohort study in Sweden has affirmed that patients diagnosed with cancer had increased risks of several common mental disorders, including depression (4). Depression can also affect the survival rate of patients with cancer. In addition, many studies have asserted that depression reduces survival in patients with cancer (5–11), although a study has presented no correlation between depression and non-small cell lung cancer (12). The possible reasons for a lower survival rate in patients with cancer and depression are non-compliance to guideline treatment (13), dysregulations of the hypothalamic–pituitary–adrenal axis, decreased immunosurveillance, and increased inflammation and oxidative stress (14, 15).

The prevalence of depression in patients with head and neck cancer is 20%–40% (9, 16, 17), which is higher than in other patients with different cancers (18). The reasons include a critical change in the facial profile and function of the patients (19–21) and radiotherapy (22). A systemic review evaluated the impacts of 77 sociodemographic, lifestyle, clinical, patient-reported outcome measures, and inflammatory factors on depression showed only depression at an earlier time point was significantly associated with the diagnosis of depression at a single (later) time point. For all other factors, evidence was inconclusive, although evidence suggests that age, marital status, education, ethnicity, hospital/region, sleep, smoking, alcohol, surgery, treatment, tumor location, and recurrence are not important associated factors.  (23)In addition, pre-op depressive symptoms can predict a longer length of hospital stay and postoperative functional status in this cancer group (24). Like the other cancers, depression before or after cancer diagnosis causes cancer progression and affects the survival of head and neck patients (9, 16, 17).

A literature review has validated that depression is strongly correlated with the cancer progression and survival of patients with head and neck cancer (9). However, no study has focused on how to improve survival in this particular group. In this study, we hypothesized that continuity of depression treatment can affect survival outcomes of head and neck cancer patients. We used a nationwide health insurance registered database to evaluate these patients. Additionally, we categorized these patients by the timing of the diagnosis of depression and the duration of depression treatment to assess the correlation between continuity of depression treatment and overall survival.

## Methods

### Study population and design

This study is a retrospective cohort study. We designed it by linking individual patient-level data to encrypted personal identification numbers from computerized data from the Cancer Registration System (CRS) database, Taiwan’s National Health Insurance Research Database (NHIRD), and the National Register of Deaths Database. The CRS began in 1979. For hospitals with more than 50 beds, specific cancer cases must be notified and collected by the Cancer Registration Center. The NHIRD is the database of the universal health insurance of Taiwan, which was started in 1995. The contents of the database include the medical orders, medical treatment, medication, and disposal for the outpatient clinic, emergency room, and hospitalized patients. The rate of insurance coverage is over 99% of the population. Every hospital in Taiwan was enrolled in the health insurance system. A detailed introduction of Taiwan’s healthcare system is described in this reference (25). The National Register of Deaths Database has been collected since 1952. All the Taiwanese nationals who issued death certificates were required to report this in the database. In this study, the patient data were collected from 2007 to 2017.

### Register linkage

Patients with the International Classification of Diseases for Oncology (ICD-O-3) (C00, 01, 02, 03, 04, 05, 06, 09, 10, 12, 13) in the cancer registration file in the CRS database, which codes for head and neck cancer, were collected. After the exclusion of patients with unknown staging and missing data, a total of 55,069 patients were enrolled. The first date of the diagnosis of head and neck cancer was defined as the index date, and the maximum follow-up time of the patients was set to 10 years. After linking with the NHIRD, we divided the patients into four groups. The patients who had no diagnosis of depressive disorder were included in the “no depression” group. A diagnosis of depressive disorder was identified by the International Classification of Diseases 9 codes (ICD-9 codes) in inpatient and outpatient claim data that included mainly two groups—major depressive disorders (MDD) (296.2, 296.3, 296.5, 296.6) and non-MDD (296.80, 296.82, 296.90, 298.0, 300.4, 309, 311). The patients who were diagnosed with depressive disorder and followed up in the psychiatric clinic only before the index day were included in the “pre-cancer only” group. The patients who were diagnosed with depressive disorder after the index date were included in the “post-cancer only” group. Further, the patients who were diagnosed with depressive disorders before the index date and followed up in the psychiatric clinic before and after the index date were included in the “both before and after cancer” group. The cohort was then linked to the National Register of Deaths Database to see whether there were differences in the survival rates among the four groups.

### Statistical analysis

A chi-square test compared the differences in variables among the four groups. Moreover, the Cox proportional hazard model was used to estimate the hazard of death with a 95% confidence interval (CI) for the four groups of patients, including crude and adjusted Cox proportional hazard models to control for confounding factors. R 4.0.2 was used for the analysis of data.

## Results

After the exclusion, our study population comprised 55,069 patients, of whom 6,345 (11.52%) were diagnosed with depression. Among these patients with depression, 2,374 (4.31%) had depression treatment before the diagnosis of head and neck cancer; 1,510 (2.74%) had depression treatment both before and after the diagnosis of head and neck cancer; and 2,461 (4.47%) had depression treatment after the diagnosis of head and neck cancer. In this study population, 50,294 (92.08%) were males, and 47,903 (87.70%) had squamous cell carcinomas. The cumulative survival rate was 56.5%, and suicide occurred in 313 (0.57%) cases (**Table 1**).

**Table 1:** Profile of the study sample by depression status.

Psychiatric outpatient care for depression									
Characteristics	No depression		Pre-canceronly		Both beforeand after cancer		Post-cancer only		*p* value
	N	%	N	%	N	%	N	%	
Total	48724		2374		1510		2461		
Age									<0.001
<60	30835	63.29%	1347	56.74%	992	65.70%	1763	71.64%	
60-75	13861	28.45%	709	29.87%	388	25.70%	571	23.20%	
>=75	4028	8.27%	318	13.40%	130	8.61%	127	5.16%	
Gender									<0.001
Male	44740	91.82%	2031	85.55%	1308	86.62%	2215	90.00%	
Female	3984	8.18%	343	14.45%	202	13.38%	246	10.00%	
Site									<0.001
Lip	1902	3.90%	80	3.37%	60	3.97%	79	3.21%	
Tongue	5075	10.42%	261	10.99%	179	11.85%	284	11.54%	
Mouth floor	11164	22.91%	735	30.96%	385	25.50%	594	24.14%	
Oropharynx	15837	32.50%	586	24.68%	458	30.33%	850	34.54%	
Pharynx, hypopharynx, larynx	12740	26.15%	595	25.06%	380	25.17%	619	25.15%	
Others	2006	4.12%	117	4.93%	48	3.18%	35	1.42%	
Stage									<0.001
1and 2	21040	43.18%	989	41.66%	772	51.13%	1050	42.67%	
3and 4	27684	56.82%	1385	58.34%	738	48.87%	1411	57.33%	
Medical center									<0.001
Yes	30738	63.09%	1255	52.86%	1066	70.60%	1837	74.64%	
No	17986	36.91%	1119	47.14%	444	29.40%	624	25.36%	
Tumor histology									0.465
Squamous cell carcinoma	42384	86.99%	2047	86.23%	1312	86.89%	2160	87.77%	
Others	6340	13.01%	327	13.77%	198	13.11%	301	12.23%	
Surgery									<0.001
Yes	22185	45.53%	1286	54.17%	701	46.42%	900	36.57%	
No	26539	54.47%	1088	45.83%	809	53.58%	1561	63.43%	
Reconstruction									<0.001
Yes	27414	56.26%	1585	66.76%	920	60.93%	1137	46.20%	
No	21310	43.74%	789	33.24%	590	39.07%	1324	53.80%	
Radiation									<0.001
Yes	17095	35.09%	883	37.19%	569	37.68%	682	27.71%	
No	31629	64.91%	1491	62.81%	941	62.32%	1779	72.29%	
Charlson Comorbidity Index score									<0.001
0	24387	50.05%	824	34.71%	463	30.66%	1160	47.14%	
1	13151	26.99%	616	25.95%	454	30.07%	693	28.16%	
>=2	11186	22.96%	934	39.34%	593	39.27%	608	24.71%	
Income differential									<0.001
<25%	11878	24.38%	657	27.67%	437	28.94%	664	26.98%	
25%~50%	9023	18.52%	251	10.57%	267	17.68%	595	24.18%	
50%~75%	15113	31.02%	882	37.15%	434	28.74%	599	24.34%	
>75%	12710	26.09%	584	24.60%	372	24.64%	603	24.50%	
Outcome									<0.001
Alive	27604	56.65%	1280	53.92%	760	50.33%	1233	50.10%	
Death	21120	43.35%	1094	46.08%	750	49.67%	1228	49.90%	
Suicide									<0.001
Yes	228	0.47%	16	0.67%	22	1.46%	47	1.91%	
No	48496	99.53%	2358	99.33%	1488	98.54%	2414	98.09%	

### Psychiatric outpatient care for depression and death

The multivariable cox analyses confirmed that the “pre-cancer only” group had worse overall survival [fully adjusted model: hazard ratio (HR) = 1.18; 95% CI = 1.11–1.25] than the “no depression” group. (**Table 2**) Moreover, the “post-cancer only” group exhibited better overall survival (fully adjusted model: HR = 0.88; 95% CI = 0.83–0.94) than the “no depression” group. In addition, patients with a diagnosis of depression had increased HRs for death by suicide compared with those in the “no depression” group (**Table 2**).

**Table 2:** Multivariable cox analyses of the association between psychiatric outpatient care for depression and overall survival.

	Not adjusted		Model 1		Model 2	
	HR	95% CI	HR	95% CI	HR	95% CI
Death						
No depression	1.00		1.00		1.00	
Pre-cancer only	1.34^a^	1.26–1.42	1.20^a^	1.15–1.30	1.18^a^	1.11–1.25
Both before and after cancer	1.07^b^	0.99–1.15	0.98^b^	0.91–1.06	1.02^b^	0.95–1.10
Post-cancer only	0.93^c^	0.88–0.98	0.91^c^	0.86–0.97	0.80^a^	0.83–0.94
Suicide						
No depression	1.00		1.00		1.00	
Pre-cancer only	1.88^c^	1.13–3.12	1.82^c^	1.09–3.03	1.79^c^	1.07–2.98
Both before and after cancer	2.89^a^	1.87–4.48	2.70^a^	1.75–4.24	2.80^a^	1.81–4.38
Post-cancer only	3.22^a^	2.35–4.41	3.10^a^	2.28–4.30	3.10^a^	2.27–4.29

Model 1: adjusted for age at diagnosis and gender and income differential and Charlson Comorbidity Index scores and medical center; Model 2: also adjusted for tumor site and reconstructive surgery and tumor stage.

HR, hazard ratio; CI, confidence interval.

a The P value is <.001.

b The P value is ≥.05.

c The P value ranges from ≥.001 to <.05.

### Depression and early and advanced stages of head and neck cancer

We divided the patients with cancer into the early (stages 1 and 2) and advanced (stages 3 and 4) stages and evaluated the influence of depression on overall survival. The “pre-cancer only” group had worse overall survival than the “no depression” group both in patients with early- and advanced-stage head and neck cancer. Additionally, the “pre-cancer only” group had worse overall survival (HR = 1.50; 95% CI = 1.32–1.70) than the “no depression” group in patients with early-stage head and neck cancer. The “post-cancer only” group also had better overall survival (HR = 0.78; 95% CI = 0.73–0.84) than the “no depression” group in patients with advanced-stage head and neck cancer (**Table 3**).

**Table 3:** Depression and overall survival of patients with head and neck cancer.

Stages 1 and 2			
Outcome	Psychiatric outpatient care for depression	HR	95% CI
Death	No depression	1.00	
	Pre-cancer only	1.50^a^	1.32–1.70
	Both before and after cancer	1.44^a^	1.27–1.62
	Post-cancer only	1.26^a^	1.13–1.39
Stages 3 and 4			
Outcome	Psychiatric outpatient care for depression	HR	95% CI
Death	No depression	1.00	
	Pre-cancer only	1.30^a^	1.21–1.39
	Both before and after cancer	1.04^b^	0.95–1.13
	Post-cancer only	0.78^a^	0.73–0.84

HR, hazard ratio; CI, confidence interval.

a The P value is <.001.

b The P value is 0.455.

### Cumulative mortality of head and neck cancer patients

**Figure 1:**
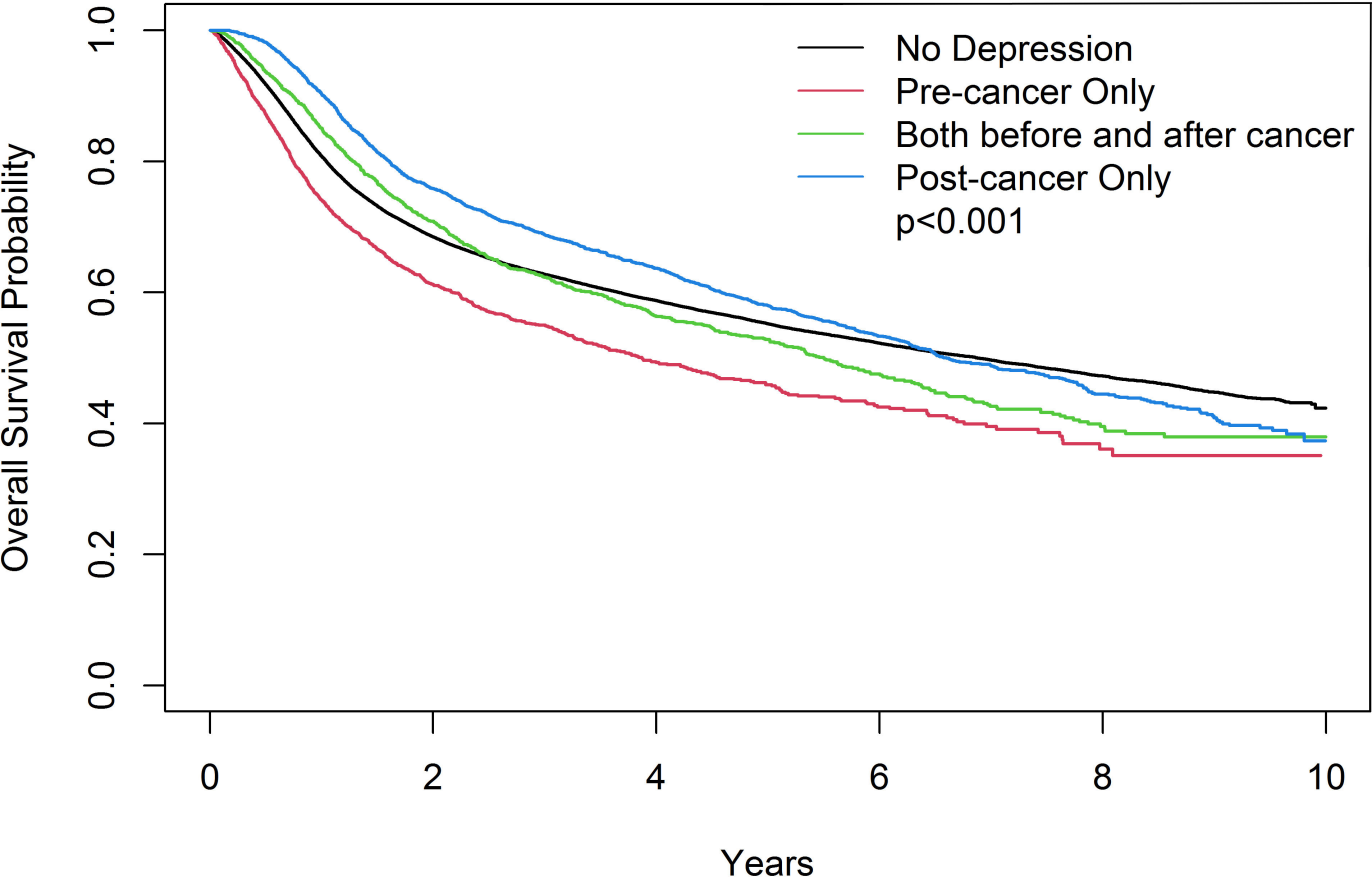
Kaplan-Meier estimates of overall survival of the head and neck cancer patients in different subgroups. The x-axis indicates years after head and neck cancer diagnosis.

The “Pre-cancer only” group had a significantly higher cumulative mortality rate than the other groups (**Figure 1**). The “Post-cancer only” group had a significantly lower cumulative mortality rate than the other groups in advanced-stage head and neck cancer.

### Continuity and frequency of psychiatric outpatient treatment for depressive disorder and overall survival

For the patients with head and neck cancer who had a diagnosis of depression before cancer, the continuity of psychiatric outpatient treatment for the depression after cancer diagnosis helped them achieve better overall survival (HR = 0.78; 95% CI = 0.71–0.86) compared with patients who had depression treatment interruptions. In the group of "both before and after cancer," patients who had an increased frequency of psychiatric outpatient clinic treatment after cancer diagnosis compared to the pre-cancer period had improved overall survival (HR =0.97; 95% CI = 0.97–0.98) (**Table 4**).

**Table 4:** Continuity and frequency of psychiatric outpatient care for depression and overall survival.

Group	Outcome	Risk	HR	95% CI
Depression before cancer	Death	Pre-cancer only	1.00	
		Both before and after cancer	0.78^a^	0.71–0.86
Depression both before and after cancer	Death	Frequency of psychiatric outpatient care for depression after cancer	0.97^a^	0.97–0.98

HR, hazard ratio; CI: confidence interval.

a The P value is <.001.

## Discussion

Depression is common in head and neck cancer patients, and the incidences vary in different studies (9, 16, 17). In this study, the incidence of depression before and after cancer was 11.52%, which is much lower than what has been previously reported, possibly because this study was performed retrospectively; the diagnosis of depression was mostly made when patients visited psychiatrists in the clinic, thereby resulting in an underestimate of the number of actual diagnoses.

In this cohort, we divided the patients with head and neck cancer into four groups according to the timing of the depression diagnosis and the interval of the treatment. Overall, patients with pre-cancer depression displayed worse outcomes compared with patients without depression. This may be a result of various factors. First, it is similar to a previously reported national database study evaluating the impact of depression on the survival of patients with breast cancer. The authors reported that women previously treated for depression had lower overall and disease-specific survival and suggested that this subgroup of patients had an increased risk of not complying with recommended breast cancer treatment (12). This thought is in line with another study published on the same topic in which patients diagnosed with depression less frequently complied with treatment (59.7% vs. 66.2%, P <.0001) (5).

The worst survival outcome in this study was observed in patients with pre-cancer depression without further psychiatric clinic visits after a cancer diagnosis compared with other groups. Our hypothesis is supported by the evidence shown in **Table 4**. Indeed, we also found that patients who had pre-cancer depression but still were regularly followed up at psychiatric clinics after their cancer diagnosis had better survival outcomes (HR = 0.78; 95% CI = 0.71–0.86). The results indicated that patients who had pre-cancer depression but continued to receive treatment in psychiatric clinics had better outcomes. In addition, the results emphasized that psychiatric intervention in patients with cancer is essential. Although one meta-analysis showed no clear evidence indicating the effect of antidepressants on the treatment of depression in people with cancer (17), our study strongly suggested that psychiatric clinic visits had a positive impact on survival outcomes.

In this study, we also found that head and neck cancer patients diagnosed with depression after cancer diagnosis exhibited good survival outcomes comparable with the “no depression” group (adjusted HR = 0.88; 95% CI = 0.83–0.94). For the subgroup analysis (**Table 3**), we found that the protective effect of treating depression in patients with advanced-stage head and neck cancer (stages 3 and 4) was more prominent than in patients with early-stage head and neck cancer (stages 1 and 2). The results of this study are not compatible with the previous study (10), which has indicated that post-op depression had poor survival outcomes. Nonetheless, this study is a retrospective study, and not every patient with depression was formally diagnosed by a psychiatrist. A patient who went to visit a psychiatrist after a cancer diagnosis may have had strong family support or was receiving cancer treatment in a medical center where psychiatric consultation was available. As such, we cannot infer that post-op depression improves survival; rather, it may be due to patient compliance with treatment guidelines, which can result in better survival outcomes.

Moreover, an advanced stage of head and neck cancer refers to the stage where patients require adjuvant therapies, including radiotherapy and chemotherapy, and may need repeated surgery. Patients in this group may also stick to the treatment protocol and thus have better outcomes. A previous study has corroborated that for patients with breast cancer, pre-cancer depression results in nonadherence to the suggested adjuvant systemic therapy (13); hence, they have poorly reported survival outcomes. Another hypothesis that supports our results is that some patients with cancer may have undetected depression and anxiety. The prevalence of major depression in patients with cancer is nearly 13%–50% on average. Li et al. affirmed that the depression rate according to the self-rating depression scale in patients with cancer was 53.0% and that the anxiety level according to a self-rating anxiety scale in patients with cancer was 32.7% (26). In a study of 903 patients with cancer, the prevalence of psychiatric disorders in a cancer hospital was 53% (major depression in 22%) (27). These numbers are higher than the depression rate reported in our study (11.52% of the cancer population) and previous studies (9.6%) (28). The results further emphasize that depression is a frequently occurring underlying disease, which is not commonly investigated and, therefore, is not diagnosed. Many patients would benefit from a formal diagnosis of depression and treatment, but they either do not have the chance or tend to neglect or ignore their condition. The identification of psychiatric morbidity in patients with cancer leads to the effective treatment of psychiatric disorders. Psychological intervention is necessary for patients with cancer, with the appropriate treatment modalities to improve their mood and quality of life. As such, psychosocial oncology is an upcoming area of interest, which should be incorporated into every multidisciplinary team that deals with cancer patient treatment (29, 30).

The suicide rate in this cohort was 0.57%. **Table 2** showed the highest hazard ratio was noted in the post-cancer depression group. A previous study showed that suicidal ideation might be associated with body image concerns (31). This may be the reason patients without predisposing depression but with post-cancer depression had the highest suicidal rate.

The limitations of our study included its retrospective nature, and the data are obtained from a single course—the National Health Insurance claims data. The diagnosis of head and neck cancer and treatment is accurate, but the number of patients with depression is underestimated as not everyone with depression is treated by a psychiatrist. Moreover, when a patient was diagnosed with depression by a psychiatrist, we couldn’t be sure that every patient has had an evaluation and fits the criteria of depression suggested by the DSM-IV manual. We also couldn’t know how psychiatrists performed psychiatric support to patients. The other limitation of this study was that we couldn’t have all the possible risk factors because this study was based on claims data. Some risk factors like tobacco usage and alcohol consumption were not collected in the database. Finally, there was no data on cancer-specific mortality from the National Health Insurance claims data, therefore some of the patients didn’t die because of cancer. Despite the study’s limitations, this study emphasizes the importance and necessity for head and neck cancer patients to receive psychiatric support, especially those with predisposing depression. Further prospective studies with explicit inclusion and exclusion criteria are needed to interpret better the correlation between head and neck cancer and depression.

In conclusion, in this nationwide cohort study of patients with head and neck cancer, we found that patients with pre-cancer depression had reduced survival outcomes, especially those who did not receive psychiatric support after their cancer diagnosis. After cancer diagnosis, patients who are experiencing clinical depression must be diagnosed. It may be beneficial for these patients to receive psychiatric support because it may improve their overall survival outcomes.

## Data availability statement

The raw data supporting the conclusions of this article will be made available by the authors, without undue reservation.

## Ethics statement

The studies involving human participants were reviewed and approved by Chang Gung Medical Foundation Institutional Review Board. Written informed consent for participation was not required for this study in accordance with the national legislation and the institutional requirements.

## Author contributions

Conceptualization: R-WH and H-KK. Data curation: Y-JL. Formal analysis: R-WH and Y-JL. Funding acquisition: C-JC and H-KK. Methodology: K-PC. Project administration: H-KK. Software: Y-JL. Supervision: K-PC and C-JC. Validation: C-JC. Writing – original draft: R-WH, FM and CYYL. All authors contributed to the article and approved the submitted version.

## Funding

This research was funded by grant CIRPD1D0033 from Chang Gung Memorial Hospital.

## Conflict of interest

The authors declare that the research was conducted in the absence of any commercial or financial relationships that could be construed as a potential conflict of interest.

## Publisher’s note

All claims expressed in this article are solely those of the authors and do not necessarily represent those of their affiliated organizations, or those of the publisher, the editors and the reviewers. Any product that may be evaluated in this article, or claim that may be made by its manufacturer, is not guaranteed or endorsed by the publisher.
